# Age- and Gender-related Disparities in Primary Percutaneous Coronary Interventions for Acute ST-segment elevation Myocardial Infarction

**DOI:** 10.1371/journal.pone.0137047

**Published:** 2015-09-09

**Authors:** Thomas Pilgrim, Dik Heg, Kali Tal, Paul Erne, Dragana Radovanovic, Stephan Windecker, Peter Jüni, AMIS Plus Investigators

**Affiliations:** 1 Department of Cardiology, Swiss Cardiovascular Center, Bern University Hospital, Bern, Switzerland; 2 Institute of Social and Preventive Medicine (ISPM) and Clinical Trials Unit, Department of Clinical Research, University of Bern, Bern, Switzerland, Switzerland; 3 Division of Cardiology, St. Anna Hospital, Lucerne, Switzerland; 4 AMIS Plus Data Center, Institute for Social and Preventive Medicine, University of Zurich, Zurich, Switzerland; 5 Institute Primary Care and Clinical Epidemiology, University of Bern, Bern, Switzerland; S.G. Battista Hospital, ITALY

## Abstract

**Background:**

Previous analyses reported age- and gender-related differences in the provision of cardiac care. The objective of the study was to compare circadian disparities in the delivery of primary percutaneous coronary intervention (PCI) for acute myocardial infarction (AMI) according to the patient’s age and gender.

**Methods:**

We investigated patients included into the Acute Myocardial Infarction in Switzerland (AMIS) registry presenting to one of 11 centers in Switzerland providing primary PCI around the clock, and stratified patients according to gender and age.

**Findings:**

A total of 4723 patients presented with AMI between 2005 and 2010; 1319 (28%) were women and 2172 (54%) were ≥65 years of age. More than 90% of patients <65 years of age underwent primary PCI without differences between gender. Elderly patients and particularly women were at increased risk of being withheld primary PCI (males adj. HR 4.91, 95% CI 3.93–6.13; females adj. HR 9.31, 95% CI 7.37–11.75) as compared to males <65 years of age. An increased risk of a delay in door-to-balloon time >90 minutes was found in elderly males (adj HR 1.66 (95% CI 1.40–1.95), p<0.001) and females (adj HR 1.57 (95% CI 1.27–1.93), p<0.001), as well as in females <65 years (adj HR 1.47 (95% CI 1.13–1.91), p = 0.004) as compared to males <65 years of age, with significant differences in circadian patterns during on- and off-duty hours.

**Conclusions:**

In a cohort of patients with AMI in Switzerland, we observed discrimination of elderly patients and females in the circadian provision of primary PCI.

## Introduction

The mortality risk of patients with acute ST-segment elevation myocardial infarction (STEMI) is a function of comorbid conditions and timely reperfusion. Guidelines speed health professionals through the process of quick diagnosis and effective treatment. When access to resources is not an obstacle, patients have the right to expect doctors to adhere to recommended standards for provision of care. If deviating from those protocols is not a medical necessity, and if a detectable pattern of deviation appears to favor one group over another, the question of discrimination may be raised [1].

A series of reports indicated discrimination in the provision of medical care for cardiac conditions. Women were less likely to be admitted to the coronary care unit [2], and were less likely to undergo invasive procedures including coronary angiography, revascularization and coronary bypass grafting [3–5]. The disparity in treatment grew greater as a woman aged [2,6]. Findings that confirmed sex discrimination were supported by those that also found ageism [7] in the treatment of cardiac patients. In many contexts, in many regions, and in many facilities, women were not receiving treatment equal to their male counterparts, even when physiological differences in their conditions and other factors, like socio-economic status and education, were accounted for.

The objective of the present analysis was to investigate age- and gender related differences in the provision of primary percutaneous coronary intervention (PCI) among patients with STEMI, focusing on circadian differences in the delivery of recommended care.

## Methods

Since 1997, the prospective Acute Myocardial Infarction in Switzerland (AMIS) registry has included patients with acute myocardial infarction (10). The registry was approved by the Over-Regional Ethical Committee for Clinical Studies and the Swiss Board for Data Security (*Commission d’experts du secret professionnel en matière de recherche médicale 1*.*05*.*01*.*10*.*-40 on May 27*, *1998)*. Among 66 participating centers, 11 sites provide primary PCI around the clock. Anonymised data are recorded on a standardized case report form. The Institute of Social and Preventive Medicine at the University of Zurich, Switzerland, manages the central database. Primary PCI had been adopted as the preferred therapeutic strategy for the treatment of STEMI, as described in the 2003 ESC guidelines [8]. For the purpose of the present analysis, we used data from patients included in the registry between January 1, 2005 and December 31, 2010.

### Ethics Statement

The study was approved by the Over-Regional Ethical Committee for Clinical Studies and the Swiss Board for Data Security (*Commission d’experts du secret professionnel en matière de recherche médicale 1*.*05*.*01*.*10*.*-40 on May 27*, *1998)*. Follow-ups were approved by all Cantonal Ethic Commissions in 2005. Data collection is conducted in accordance with the EU Note for Guidance on Good Clinical Practice CPMP/ECH/135/95 and the Declaration of Helsinki. The AMIS Plus project is registered at ClinicalTrials.gov Identifier: NCT01305785. The study complied with the Declaration of Helsinki. Written informed consent as given by the patients for their information to be stored in the hospital database and used for research.

### Patient Selection and Definitions

STEMI was defined as chest pain with an onset <12 hours prior to presentation, and by new ST-segment elevation by >1mm in ≥2 contiguous leads, or new left bundle branch block in combination with cardiac biomarkers (CK and CK-MB) elevated to at least twice the upper limit of normal. Patients were included if they satisfied our definition of STEMI and primarily presented to one of the 11 sites with 24h primary PCI facilities. Patients who were transferred from referring hospitals were excluded. Patients were stratified according to gender and age <65 or ≥65 years.

### Statistical Analysis

We compared the in-hospital management of young women, elderly men and elderly women, to young men. Baseline characteristics are presented as counts with percentages (p-values from chi-square-tests), and means ± standard deviations (p-values from t-tests or Fisher’s exact test). Pain onset to hospital presentation, and door-to-balloon times are presented as medians (both in minutes), and 25%-75% interquartile ranges (p-values from Mann-Whitney U-tests). We used logistical regression to compare in-hospital management of the four groups (no PCI received, and door-to-balloon time of more than 90 minutes). We present crude and adjusted odds ratios OR with 95% confidence interval CI and p-values for young women, elderly men and elderly women as compared with young men. Adjustments were made for sinus rhythm, chest pain, dyspnea, Killip class and resuscitation at hospital admission, using multiple imputation for missing covariates (n = 10 data-sets generated). P-values of the interaction effect of age with gender are also presented. Door-to-balloon times in minutes were log-transformed and related to the clock-time of hospital admission (0 to 24 hours) using trigonometric regression models [9]. Analyses were performed crude and adjusted for above-mentioned covariates at hospital admission using Inverse-probability weighing. Inverse-probability of age and gender were computed using a full-factorial propensity score derived from covariates at hospital admission, again after multiple imputation of missing values (n = 10 data-sets generated). All statistical analyses were performed with Stata 12.1 (StataCorp, Texas, USA).

## Results

**Fig 1:**
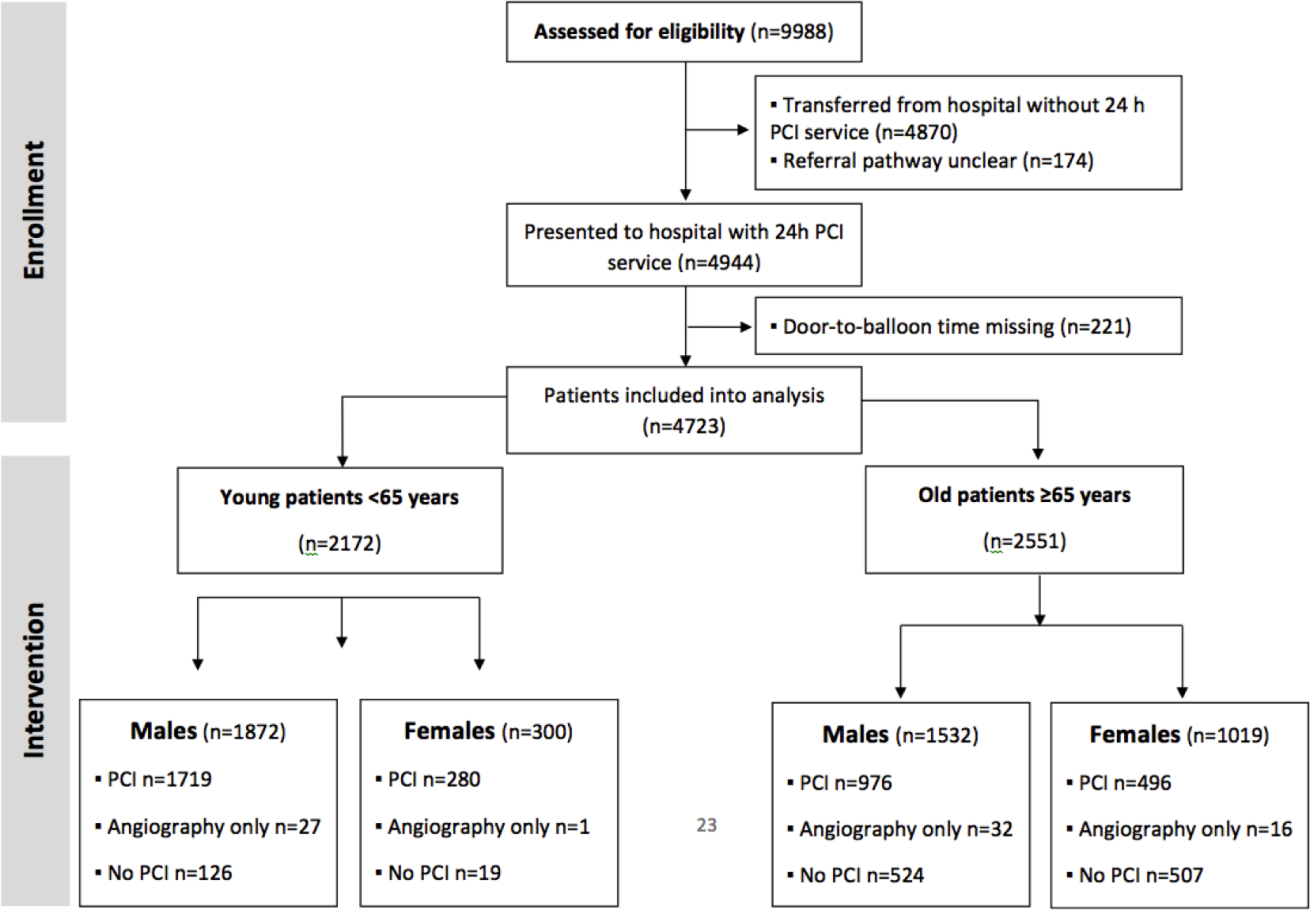
Flow diagram. Flow diagram of patients included into the analysis.

The AMIS registry included 9,988 patients who presented to primary, secondary and tertiary care hospitals in Switzerland with STEMI, between January 1, 2005 and December 31, 2010 (**Fig 1**). 4,944 patients directly attended the emergency room of one of the 11 sites with 24h primary PCI facility, 221 patients were excluded due to missing door-to-balloon times, the remaining 4723 were included in our study (95.6%). Baseline characteristics, stratified by age and gender, are summarized in **Table 1**. Women accounted for 28% of patients admitted with STEMI; 54% of patients were ≥65 years of age. Even after stratification for age, female patients were older than their male counterparts (p<0.001), had a lower body mass index (p<0.001), and more often a history of hypertension (p<0.001) and diabetes (p<0.001). Men had more frequently a diagnosis of dyslipidemia (p = 0.009).

**Table 1:** Baseline characteristics of all patients.

	Young <65 Years			Old ≥65 Years			Old vs Young	Female vs Male
	Male	Female	p-value	Male	Female	p-value	p-value	p-value
	N = 1872	N = 300		N = 1532	N = 1019			
Age, (years)	53.6 ± 7.8	56.0 ± 7.4	<0.001	76.5 ± 7.5	80.5 ± 7.6	<0.001	<0.001	<0.001
BMI (kg/m^2^)	27.2 ± 4.1	26.8 ± 5.6	0.205	26.4 ± 3.9	25.4 ± 5.1	<0.001	<0.001	<0.001
***Cardiovascular Risk Factors***		*** ***	*** ***	*** ***	*** ***	*** ***	*** ***	*** ***
Hypertension, n(%)	783 (44%)	143 (50%)	0.064	986 (69%)	739 (77%)	<0.001	<0.001	<0.001
Current smoker, n(%)	1064 (60%)	175 (61%)	0.948	280 (21%)	120 (14%)	<0.001	<0.001	<0.001
Dyslipidemia, n(%)	868 (51%)	135 (49%)	0.559	677 (51%)	371 (46%)	0.011	0.333	0.009
Diabetes mellitus, n(%)	238 (13%)	51 (18%)	0.054	322 (22%)	238 (25%)	0.139	<0.001	<0.001
Prior MI or stable angina, n(%)	437 (24%)	69 (23%)	1.000	607 (40%)	366 (36%)	0.055	<0.001	0.132
***Clinical Presentation***								
Heart rhythm			0.182			0.621	<0.001	<0.001
Sinus rhythm, n(%)	1786 (95%)	279 (93%)	0.082	1308 (85%)	867 (85%)			
Atrial fibrillation, n(%)	26 (1%)	6 (2%)	0.435	126 (8%)	93 (9%)			
Other rhythms, n(%)	59 (3%)	15 (5%)	0.120	98 (6%)	59 (6%)			
Chest pain, n(%)	1677 (94%)	274 (92%)	0.301	1265 (87%)	801 (82%)	0.001	<0.001	<0.001
Dyspnea, n(%)	359 (22%)	68 (26%)	0.236	494 (36%)	373 (40%)	0.065	<0.001	<0.001
Killip’s classification, n(%)			0.454			0.002	<0.001	<0.001
I	1629 (87%)	255 (85%)	0.355	1033 (68%)	630 (62%)			
II	117 (6%)	26 (9%)	0.131	310 (20%)	267 (26%)			
III	29 (2%)	5 (2%)	0.803	90 (6%)	68 (7%)			
IV	92 (5%)	13 (4%)	0.772	91 (6%)	50 (5%)			
Resuscitation, n(%)	170 (9%)	20 (7%)	0.187	92 (6%)	39 (4%)	0.014	<0.001	<0.001

Depicted are counts (%, p-values from chi square or Fisher's tests), means ± standard deviations (p-values from t-tests).

### Symptoms and Presentation

Elderly patients and women were less likely to present with chest pain (p<0.001) and more likely to complain of shortness of breath (p<0.001). More time elapsed between the onset of their symptoms and presentation to the hospital (males <65 years: median 129 min [interquartile range 76 to 275]; females <65 years: 180 min [105 to 380]; males ≥65 years: 175 min [96 to 450]; females ≥65 years 195 min [111 to 493], p<0.001 for difference between groups). Elderly patients and males were more often admitted with cardiogenic shock/after resuscitation than were younger patients and females (p<0.001); this was driven by a differential between elderly males and women (p = 0.014, **Table 1**).

### In-hospital Management

**Fig 2:**
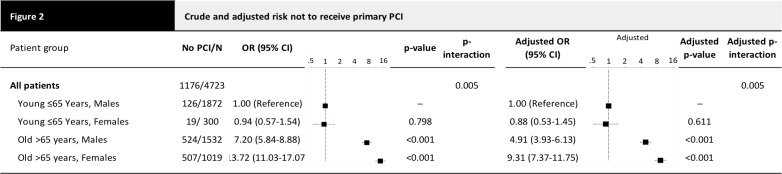
Crude and adjusted risk not to receive primary PCI. Depicted are number of patients without PCI/total number of patients, crude and adjusted odds ratios OR (with 95% confidence interval CI), and crude and adjusted p-values comparing vs young males. The ORs are adjusted for the clinical presentation parameters. P-values interaction are the p-values for the interaction effect of age x gender in the model containing the main effects and this interaction.

Procedural characteristics are summarized in **Table 2**. Younger patients were more consistently treated with aspirin and clopidogrel than were patients ≥65 years (p<0.001). Women were less likely to receive clopidogrel therapy (p<0.001); this was driven by a difference between elderly women and men (p<0.001). GpIIbIIIa inhibitors were more often administered to younger patients (p<0.001) and men (p<0.001). Patients <65 years of age were treated with primary PCI in ≥90% of cases, regardless of their gender (p = 0.434). **Fig 2**shows that elderly patients ≥65 years of age, and women in particular, were less likely to receive primary PCI (men adj. HR 4.91 [95% CI 3.93–6.13]; women adj. HR 9.31 [95% CI 7.37–11.75], p for interaction between age and gender, 0.005) than men <65 years of age.

**Table 2:** Procedural characteristics of all patients.

	Young <65 Years			Old ≥65 Years			Old vs Young	Female vs Male
	Male	Female	p-value	Male	Female	p-value	p-value	p-value
	N = 1872	N = 300		N = 1532	N = 1019			
***Reperfusion Strategy***			0.434			<0.001	<0.001	<0.001
Primary PCI, n(%)	1703 (92%)	280 (94%)	0.478	962 (64%)	492 (49%)	<0.001	<0.001	<0.001
Secondary PCI*, n(%)	16 (1%)	0 (0%)	0.150	14 (1%)	4 (0%)	0.150	1.000	0.035
Thrombolysis only, n(%)	32 (2%)	5 (2%)	1.000	32 (2%)	14 (1%)	0.224	0.825	0.326
None, n(%)	94 (5%)	14 (5%)	0.887	492 (33%)	493 (49%)	<0.001	<0.001	<0.001
***Immediate Drug Therapy***								
Aspirin, n(%)	1825 (98%)	296 (99%)	0.391	1425 (93%)	949 (94%)	0.563	<0.001	0.305
Clopidogrel, n(%)	1648 (88%)	262 (88%)	0.698	1140 (75%)	650 (64%)	<0.001	<0.001	<0.001
Unfractioned Heparin, n(%)	1502 (81%)	238 (80%)	0.752	1081 (71%)	671 (67%)	0.017	<0.001	<0.001
LMWH, n(%)	460 (25%)	80 (27%)	0.429	441 (29%)	317 (32%)	0.198	<0.001	0.011
Betablocker, n(%)	1193 (64%)	171 (58%)	0.027	864 (57%)	593 (59%)	0.344	<0.001	0.133
ACE/ARB, n(%)	1014 (55%)	152 (52%)	0.313	766 (51%)	417 (42%)	<0.001	<0.001	<0.001
GPIIb IIIa-Inhibitor, n(%)	978 (53%)	131 (44%)	0.007	480 (32%)	227 (23%)	<0.001	<0.001	<0.001
Statin, n(%)	1549 (84%)	242 (81%)	0.402	1119 (74%)	641 (64%)	<0.001	<0.001	<0.001
Length of hospital stay (days)	6.2 ± 6.7	6.7 ± 6.3	0.239	7.7 ± 6.8	9.7 ± 7.8	<0.001	<0.001	<0.001

Depicted are counts (%, p-values from chi square or Fisher's tests), means ± standard deviations (p-values from t-tests).

*PCI after fibrinolysis.

**Fig 3:**
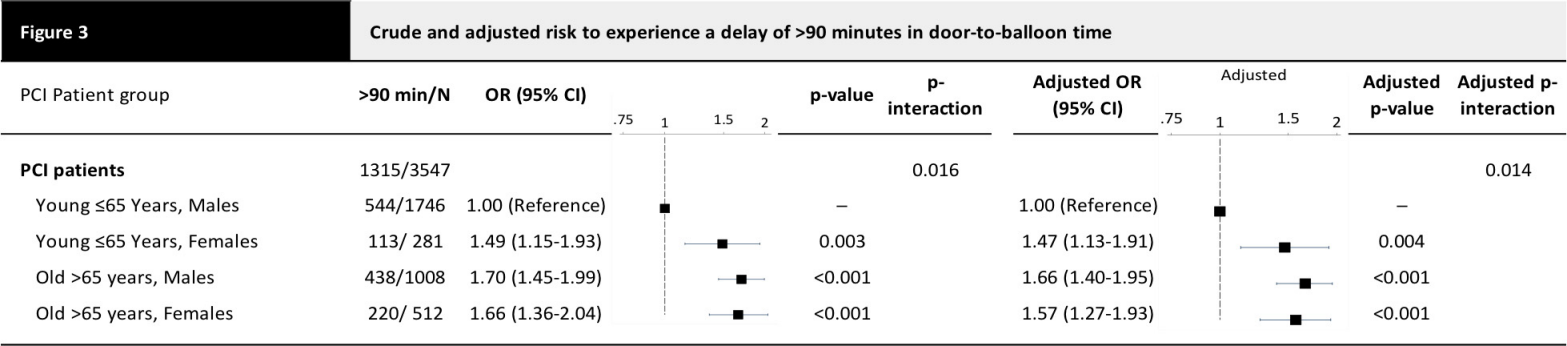
Crude and adjusted risk to experience a delay of >90 minutes in door-to-balloon time. Depicted are number of patients with >90 minutes from door to balloon/total number of patients, crude and adjusted Odds Ratios OR (with 95% confidence interval CI), and crude and adjusted p-values comparing vs young males. Adjusted for the clinical presentation parameters. P-interaction is the p-value for the interaction effect of age x gender in the model containing the main effects and this interaction.

Elderly patients and women had longer door-to-balloon times than young men <65 years of age (males <65 years: median 60 min [interquartile range 31 to 113]; females <65 years: 71 min [40 to 131]; males ≥65 years: 78 min [43 to 180], females ≥65 years: 80 min [45 to 193], p for difference between groups <0.001). Accordingly, **Fig 3**shows that men <65 years (adj HR 1.66 (95% CI 1.40–1.95), p<0.001) and women <65 years (adj HR 1.57 (95% CI 1.27–1.93), p<0.001) were more likely to have door-to-balloon time delays of more than 90 minutes (adj HR 1.47 [95% CI 1.13–1.91], p = 0.004) than were men <65 years of age.

**Fig 4:**
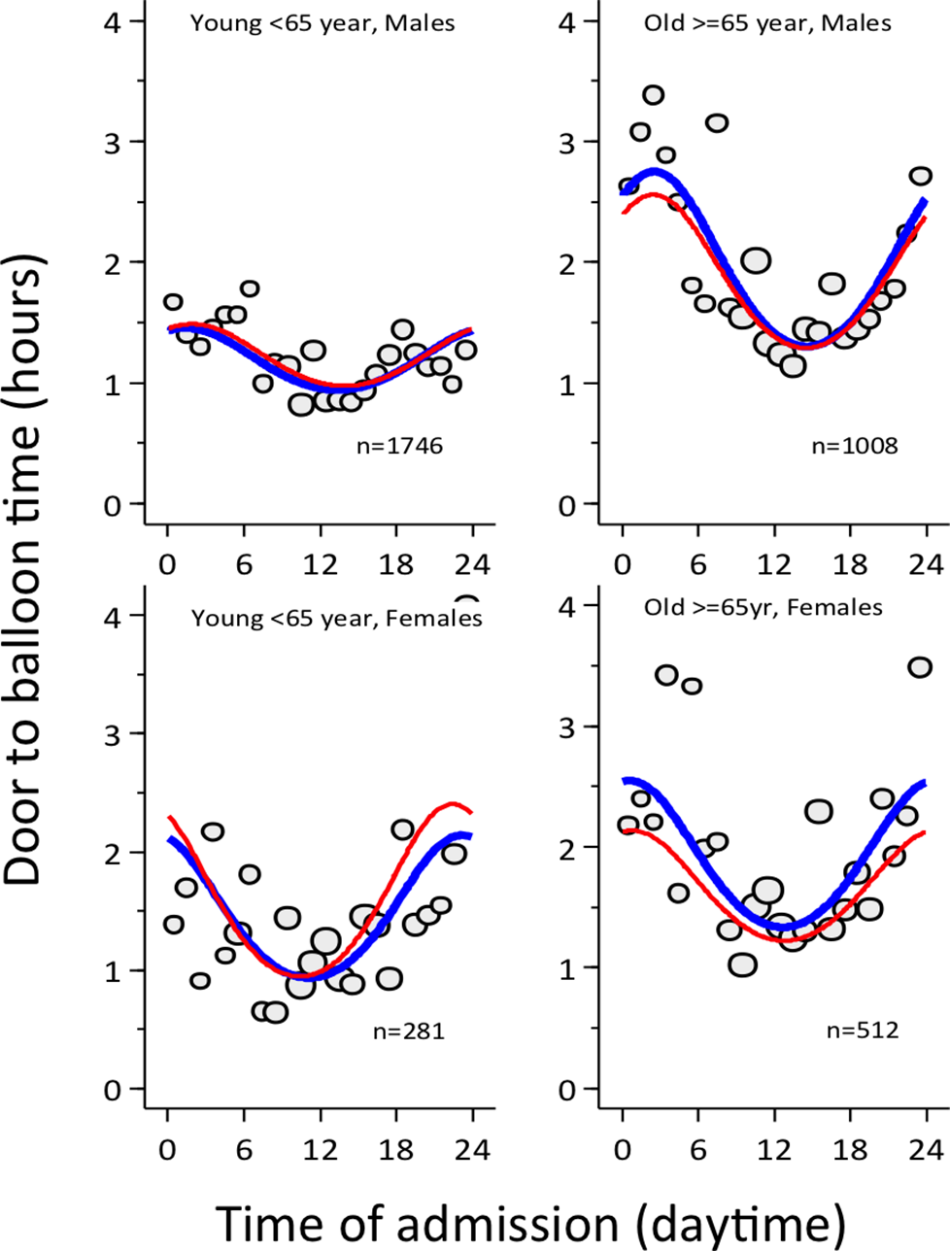
Circadian pattern of door to balloon times in women and men <65 years and ≥65 years. The x-axis shows the clock-time of hospital admission, the y-axis shows the door-to-balloon time. The red line shows the crude, the blue line the adjusted estimate.

**Fig 4**presents door-to-balloon times according to clock-time of admission to the hospital by age and gender, crude and adjusted for covariates at hospital admission. The peak delay between admission and PCI took place around midnight in all four groups. Door-to-balloon time during regular duty-hours was similar for males and females <65 years of age, but during off-hours a new pattern emerged, where longer delays were more common for women. Door-to-balloon times for elderly patients were consistently longer than for younger patients at all times, but the difference was particularly pronounced during off hours. **Table 3**presents average door-to-balloon times estimated for noon and midnight in the four groups. Both, males and females <65 years had average door to balloon times of 57 min at noon, which increased to 86 min at midnight in males, but 127 min in females. Among elderly patients ≥65 years, average door-to-balloon times were 85 and 80 min at noon and increased to 152 min at midnight in both, males and females. Estimated differences in door-to-balloon time between noon and midnight were 29 min in males <65 years, but approximately 70 min in young females and the elderly.

**Table 3:** Estimated average door-to-balloon time in minutes (95% confidence interval).

	Noon (12 AM)	Midnight (12 PM)	Difference
**Young <65 Years**			
Males	57 min (52–62)	86 min (76–97)	29 min (17–41)
Females	57 min (45–70)	127 min (91–175)	70 min (26–114 min)
**Old ≥65 Years**			
Males	85 min (74–97)	152 min (124–187)	67 min (33–101)
Females	80 min (67–96)	152 min (115–200)	72 min (27–117)

## Discussion

Our analysis shows that elderly patients with STEMI were exposed to a 5–9 fold increased risk of being withheld primary PCI as compared to patients <65 years of age. Among patients who received PCI, an increased risk of a delay in door-to-balloon time of more than 90 minutes was found in elderly males and in females irrespective of age. The elderly and women were particularly discriminated against during off hours, with delays following a circadian pattern. Young men had the shortest door-to-balloon times and were the most likely to be treated within the recommended time limits also during off hours.

Whereas well above 90% of patients <65 years of age underwent primary PCI, this proportion decreased to less than two-thirds among patients ≥65 years of age. Patients not referred for primary PCI are a composite of the ones in which interventions were deemed futile, and the ones in which incommensurate delays resulted in forfeiture of the opportune treatment window. Based on the available data, we could not differentiate between deliberate waiver of treatment and unwitting discrimination. Since the resources of Swiss hospitals are abundant, the observed age- and gender-related disparities in the provision of primary PCI for STEMI remain poorly explained. The fact that the margin of difference was smallest during working hours and highest during off-hours suggests that the decision making of hospital personnel is time-dependent. This notion is supported by an earlier study that also used data from the AMIS Plus Registry; it examined variation in thrombolysis and PCI in Swiss hospitals, and found that, overall, thrombolysis was administered more frequently during off hours, while PCI was administered more frequently during working hours [10]. In our study, however, the most important distinction is not that medical personnel are making different decisions at different times; the most important distinction is that the differences already evident in their decisions about male, female and elderly patients are exacerbated during off-duty hours.

Our data does not allow us to isolate the bottlenecks in door-to-balloon time, but we must consider that physicians and other hospital staff may slow the process down for some patients more than others. Slowdowns can occur at any stage, or at many stages. Given our results, it is reasonable to assume that, somewhere along the line, staff members are making decisions about patients based on patient group membership rather than guidelines [11,12]. There is no reason to believe this reflects a pattern of conscious intent, since many studies document the presence of unconscious bias among medical personal, and the effects it has on their decisions and actions [13].

The impulse to act on unconscious bias is heightened when physicians are under stress [14] and when personnel must deal with stressed patients. For example, a study of implicit gender bias showed that medical students and residents were most likely to make biased decisions about cardiac patients when faced with a “stressed or anxious woman with CHD-like symptoms” [15]. When clinicians are uncertain, they are also more likely to rely on subjective judgment [16]. Since the differential we found in treatment is greatest during off hours, it is possible that hospital staff members are under more stress during that period, and are forced to make decisions more quickly with less support, thus increasing their risk of bias. Reducing stress would be beneficial, but may not be possible. Training and awareness programs have had success in reducing the chance that physicians will make biased decisions [17–19]. Making staff aware of a tendency towards bias under off-duty conditions and “de-biasing” [20] might counteract this tendency.

Peterson et al analyzed data from approximately 2.5 million patients with STEMI admitted to U.S. hospitals between 1990–2006, and found that, despite very clear, and widely-adopted ACCC/AHA STEMI and NSTEMI guidelines, there were still “disparities in STEMI treatment among women, blacks, and elderly patients” and that, in some regards, these appeared to be getting worse rather than better [21]; the disparity was confirmed again by Stock et al in 2012 [22]. Results of studies conducted specifically to test adherence to and effectiveness of standardized guidelines show that standardized protocols reduce disparities and improve patient outcomes when they are in place and enforced [23–25]. Specific guidelines for the treatment of women may equalize their treatment [26], but there is evidence that even when guidelines are provided, physicians do not consistently adhere to them [27].

Blair et al suggest a course for future research. We have made some progress towards the first goal they describe, which is determining “the degree of implicit bias with regard to the full range of social groups for which disparities exist” [12] The second goal they set is “understanding the relations between implicit bias and clinical outcomes,” which requires more differentiated analysis of the data, so that we can begin to understand where the delays in door-to-balloon time are most likely to occur, and, thus, to accomplish the third goal: intervening to prevent them.

The present analysis has several limitations. First, only patients surviving to hospital admission are included into the AMIS registry, which therefore reflects a selection of patients with a more favorable clinical course in the acute phase of STEMI. Second, even though consecutive inclusion into the AMIS registry of all patients presenting with STEMI is recommended, we cannot rule out selection bias. Third, even though we performed analysis adjusted for the characteristics of clinical presentation at hospital admission, the observed disparities may reflect latent differences in baseline characteristics not adequately accounted for in the adjusted analysis. Third, we have no information on the type of stent implanted. Drug-eluting stents have demonstrated superior efficacy as compared to bare-metal stents [28], and may have contributed to the disparity in management of patients presenting with STEMI. Fourth, no information on completeness of revascularization during primary PCI was recorded in the AMIS database, which may affect clinical outcome [29]. Finally and most importantly, we did not investigate, whether the documented disparities in management had an effect on clinical outcome.

In conclusion, we observed disparities in treatment of patients with STEMI, which were greatest during hospital off hours. Recommendations for minimal door-to-balloon times continue to be more strictly followed for young men, while young women and elderly receive less timely treatment.
